# ApoA1 and ApoB Are Associated with Fracture Risk in Patients with Type 1 Diabetes

**DOI:** 10.3390/jcm15083019

**Published:** 2026-04-15

**Authors:** Emma Paulsson, Sergiu Bogdan Catrina, Cecilia Toppe, Edwin van Asseldonk, Hans J. Arnqvist, Simona I. Chisalita

**Affiliations:** 1 Department of Health, Medicine and Caring Sciences, Faculty of Medicine and Health Sciences, Linköping University, 581 85 Linköping, Sweden; 2 Department of Endocrinology in Linköping, Region Östergötland, 581 85 Linköping, Sweden; 3 Institutionen för Molekylär Medicin och Kirurgi, Karolinska Institutet, 171 76 Stockholm, Sweden; 4 Center for Diabetes, Academic Specialist Center, 113 65 Stockholm, Sweden; 5 Department of Endocrinology, Ryhov Hospital, 551 85 Jönköping, Sweden; 6 Department of Medicine, Oskarshamn Hospital, 572 51 Oskarshamn, Sweden; 7 Department of Biomedical and Clinical Sciences, Linköping University, 581 85 Linkoping, Sweden

**Keywords:** type 1 diabetes, fracture, biomarkers, ApoB, ApoA1, copeptin

## Abstract

**Background:** Individuals with type 1 diabetes (T1D) have an increased fracture risk, but no clear biomarkers have been linked to this risk. ApoA1 and ApoB were selected due to their association with metabolic disturbances in T1D. Copeptin was included given emerging evidence that ADH influences bone remodeling and glucose metabolism. The aim of this study was to identify biomarkers associated with fractures in patients with T1D. **Methods:** This prospective, population-based study included 473 individuals with T1D and 465 individuals without diabetes. Fasting blood samples were collected at baseline, and fracture outcomes were assessed after approximately 10 years. ApoA1, ApoB, CRP, GFR, copeptin, and HbA1c were analyzed. Cox regression was used to evaluate associations with fracture risk, and results were calculated per unit increase. **Results:** In total, 91 fractures occurred. A Kaplan–Meier analysis was performed to compare fracture risk between the control group and individuals with T1D. The results demonstrated a higher risk of fractures over time in patients with T1D compared to controls (*p*-value 0.037). When we divided the population by patient/control status, we found that, after adjustment for all investigated variables (HbA1c, GFR, CRP, copeptin, age, smoking, cortisone treatment, physical activity, lipid-lowering medication, and gender), both ApoA1 (HR 4.290, CI 1.871–9.837, *p*-value < 0.001) and ApoB (HR 7.625, CI 1.995–29.138, *p*-value 0.003) remained independently associated with fracture risk in the T1D group. **Conclusions:** Higher ApoA1 and ApoB levels are associated with increased fracture risk in individuals with T1D, independently of confounders. Additionally, individuals with T1D have a higher overall fracture risk compared to controls.

## 1. Introduction

Individuals with type 1 diabetes have an increased risk of fractures; nevertheless, the mechanisms underlying this association remain poorly understood, making it difficult to predict which individuals are most at risk. Several contributing factors have been proposed, such as low bone mineral density, altered bone microstructure, and diabetic complications [1]. Since the mechanisms underlying the increased fracture risk in patients with type 1 diabetes are not fully understood, it remains unclear whether conventional approaches to fracture risk prediction are applicable to this population. For example, previous research has shown that individuals with type 1 diabetes tend to have a lower trabecular bone score, suggesting alterations in bone microarchitecture that may not be fully captured by standard risk assessment tools such as the FRAX score, which relies on bone mineral density (BMD) [2].

### 1.1. Biomarkers for Predicting Fractures

Several potential biomarkers have been explored in association with the risk of developing osteoporosis and acquiring fractures for patients with type 1 diabetes, with varying results [3,4].

Apolipoprotein B (ApoB) and Apolipoprotein A1 (ApoA1) have been connected with bone metabolism [5]. Previous research has examined the relationship between ApoB and BMD, demonstrating a significant negative association with lumbar spine BMD in the general population [6]. The researchers also found that a higher concentration of ApoB increased the risk of osteoporosis. These associations were observed to be influenced by sex, age, and ethnicity [6]. ApoB has been investigated in both type 1 and 2 diabetes patients, in whom insulin is thought to decrease the concentration of ApoB [7]. Regarding ApoA1, there is conflicting data about the risk for osteoporosis. A higher ApoA1 concentration has been associated with a decreased risk of osteoporosis in type 2 diabetes patients [8], whereas in the general population, a higher concentration of ApoA1 has been linked to an increased risk of osteoporosis [9].

ApoA1 and ApoB reflect specific aspects of the metabolic system, and patients with type 1 diabetes are known to have an altered lipid profile. This imbalance has been linked to an increased risk of cardiovascular events, while its association with fracture risk remains poorly understood. Nevertheless, existing studies suggest a possible relationship between these markers and fracture risk, although the underlying mechanism has yet to be clarified [5].

Hemoglobin A1c (HbA1c) is used as a biomarker of glycemic control in individuals with diabetes. Several studies have examined the relation between HbA1c and fracture risk. In patients with type 2 diabetes, increased levels of HbA1c were associated with an increased risk of fractures [1]. There is some data suggesting that higher HbA1c levels are associated with an increased risk of fractures regardless of the type of diabetes diagnosis [10]. However, not all previous studies have found an association between fracture risk and HbA1c [1,11].

Antidiuretic hormone (ADH, or arginine vasopressin, AVP) regulates water balance and electrolytes by controlling kidney water reabsorption [12]. Because ADH cannot be reliably measured, copeptin (CT-proAVP) is commonly used as a stable surrogate [13,14,15,16]. ADH is produced from the precursor pre-pro-vasopressin, which is cleaved into three equimolar components; ADH, copeptin, and Neurophysin II. Consequently, circulating copeptin concentrations accurately mirror ADH concentrations [15].

A study on mice by Tamma et al. looked at genetically modified mice (lacking ADH receptors) to show that ADH negatively regulates osteoblasts and stimulates osteoclasts [12], suggesting that a higher level of ADH results in increased bone loss and risk of developing osteoporosis. Hyponatremia is associated with elevated ADH levels and an increased risk of bone loss [12,17]. Some argue that patients with chronic hyponatremia should, in fact, be screened for osteoporosis [17,18]. The theory behind bone loss in these patients is that sodium from the bones is released in response to the hyponatremia [12]. The study by Tamma et al. also showed that osteoblasts and osteoclasts express ADH- receptors on the cell surface. ADH deactivates osteoblasts and activates osteoclasts [12]. Interestingly, copeptin is also known to be slightly elevated among diabetics [19]. ADH is thought to play an important role in regulating glucose and fat metabolism. It increases glucose production in the liver and influences insulin or glucagon release depending on plasma glucose levels. ADH also stimulates ACTH release, increasing glucocorticoid levels, which may further disrupt metabolic control [20].

### 1.2. Aim

The aim of this population-based prospective observational study is to explore ApoA1, ApoB, and copeptin as biomarkers of future fracture risk in individuals with type 1 diabetes. By identifying reliable biomarkers, we aim to improve assessment and targeted interventions.

## 2. Materials and Methods

### 2.1. Participants

The study population in this observational prospective investigation consisted of the previously described population from the LeDIG cohort, consisting of 1481 individuals with type 1 diabetes and control individuals, recruited in cooperation with nine hospitals in the southeast region of Sweden between 2010 and 2013 [21]. Participants with type 1 diabetes were identified via the national diabetes register (NDR), and the control participants were selected from the Swedish general population by using the Swedish national population register. All the participants were recruited by means of a posted letter accompanied by a questionnaire to fill out. Participants with type 1 diabetes had an additional part to fill out. Patients were invited to participate first, and upon a positive response, a matched control was invited. If no response was received, the next eligible control was contacted. In Sweden, almost everyone with Type 1 Diabetes attends hospital outpatient diabetes clinics that use the NDR. Therefore, we hope that our study population would include almost all patients with Type 1 Diabetes in the southeastern region. Of the 1727 patients with Type 1 Diabetes who met the inclusion criteria, 773 agreed to participate. Among the controls, 721 of 1995 invited individuals participated. From the control group, 13 individuals were excluded due to fasting plasma glucose levels of ≥7 mmol/L, leaving 708 controls in the final sample. The response rate was therefore 45% among patients and 36% among controls. A previous dropout analysis among patients showed that non-participants were younger and more likely to be male [21]. Due to incomplete questionnaires and missing fasting blood samples, the analytical sample was reduced to 987 (from 1481) participants. In all, 49 participants died during our follow-up period. Participants who died prior to follow-up were excluded to ensure a consistent definition of the outcome and complete follow-up data on fracture events. While we acknowledge that time-to-death information was available, we did not model death as a censoring event or apply competing risk methods. This decision was primarily based on the limited number of fracture events relative to the number of covariates included in the fully adjusted models, which raised concerns about model stability, particularly for more complex approaches such as competing risk analyses.

**Figure 1:**
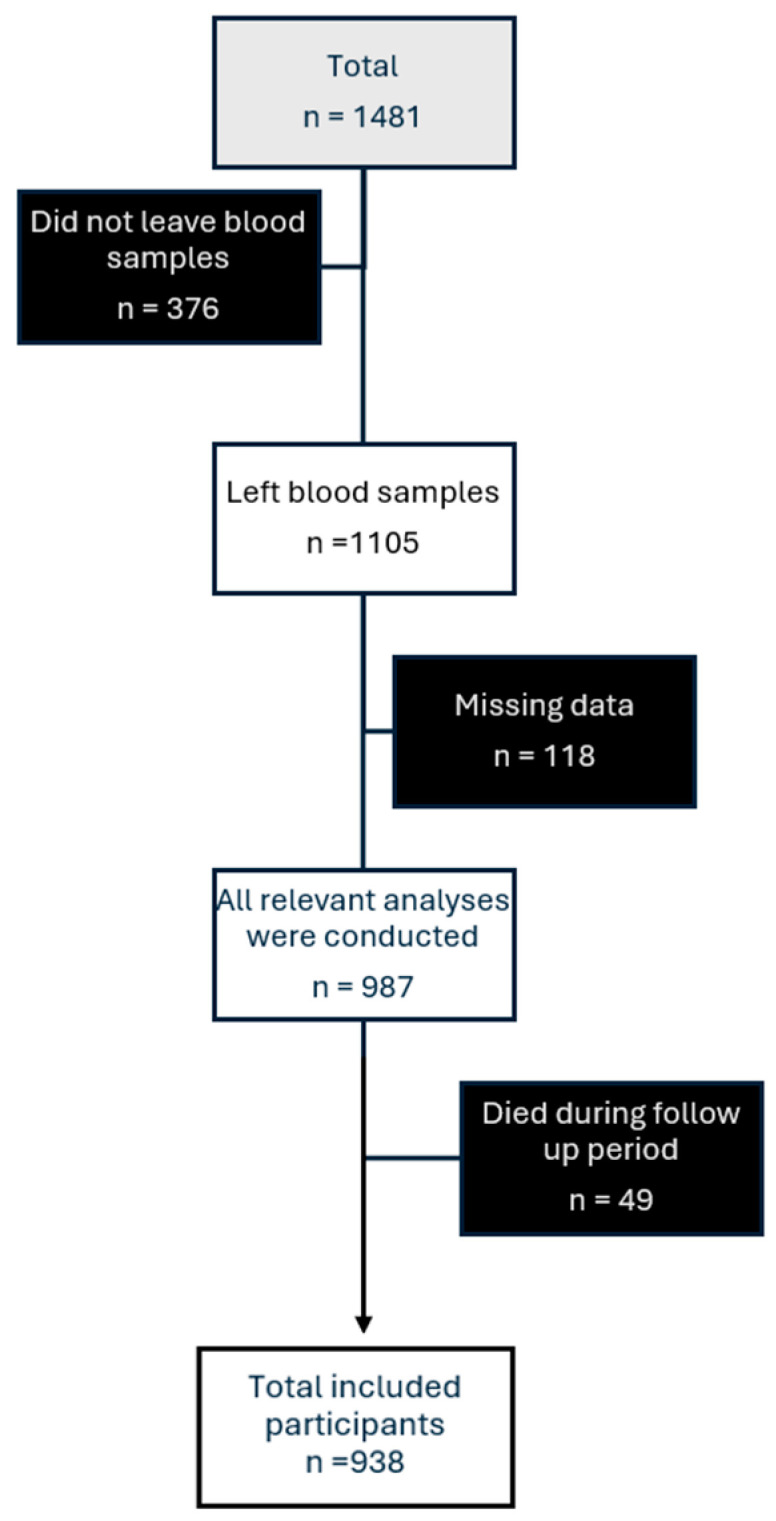
Selection of Participants. A flowchart illustrating the inclusion and exclusion process.

This approach may, however, introduce selection bias. Specifically, excluding individuals who died during follow-up is likely to lead to an underestimation of fracture risk, as those individuals may have had a higher burden of comorbidities and, consequently, a higher underlying fracture risk. As a result, the observed associations may appear weaker than they truly are, potentially underestimating the fracture risk. After exclusion, 938 participants were left (see Figure 1), of which 473 (267 women vs. 206 men) had type 1 diabetes. The remaining 465 participants in LeDIG were controls (172 men and 293 women) matched for age (±5 years), sex, and comorbidities. Six control participants were excluded from the Cox regression analysis due to missing recruitment dates. The LeDIG cohort is quite unique since the patients with type 1 diabetes had a long disease duration of over 20 years at inclusion. All participants with type 1 diabetes likely had no endogenous insulin secretion based on a long diabetes duration and unmeasurable or barely detectable C-peptide levels, characteristic of type 1 diabetes.

### 2.2. Data Collection

At recruitment, the participants filled out a questionnaire, through which various basic variables were collected. Various kinds of background information were requested in the survey [21]. The participants’ ages were determined based on their Swedish social security numbers. Some variables were recoded for analysis. Smoking status was coded as 1 for smokers and 0 for non-smokers. Physical activity was assessed with four response options based on frequency per week, defined as at least 30 min of activity (e.g., walking or equivalent, individually adapted, with all types of activity included). The responses were recoded as follows: 1 = never, 2 = less than once per week, 3 = 1–2 times per week, 4 = 3–5 times per week, and 5 = daily. Treatment with cortisone was also included and assessed using the question, “Are you currently undergoing treatment with cortisone in tablet form?” Responses were coded as 1 = yes and 0 = no. Additionally, participants were asked, “Are you currently receiving treatment for high blood lipids?” Responses were coded as 1 = yes and 0 = no.

Fasting blood samples were collected at recruitment at the local hospital, and all blood samples were analyzed with routine methods at the time at the Department of Clinical Chemistry, Linköping University Hospital. This included CRP, ApoB, ApoA1, and GFR. HbA1c was only analyzed for individuals in the type 1 diabetes group. Parts of the blood samples were frozen at −80 °C in the Biobank Facility at Linköping University Hospital for later analysis. Biomarker values were not standardized or log-transformed prior to regression analysis.

HbA1c was analyzed using a TOSHOH G7 automated hemoglobin analyzer (Tosoh Bioscience, Tokyo, Japan). High-sensitivity C-reactive protein (hsCRP) was analyzed with a detection limit of 0.3 mg/L using an Advia 1800 instrument (Siemens Healthcare Diagnostics, Deerfield, IL, USA) and a latex-enhanced immunochemistry method with reagents from the same company. ApoB and ApoA1 were measured using turbidimetry with Dako antibodies on the ADVIA 1800 analyzer.

The estimated glomerular filtration rate (eGFR) was determined using the CKD-EPI formula based on serum creatinine levels. Creatinine measurement was performed on the ADVIA 1800 analyzer (Siemens Healthcare Diagnostics, Deerfield, IL, USA) using a standardized Jaffe-based method. To enhance accuracy, the assay contained a rate-blanking measurement to compensate for interference from bilirubin and a correction for interception due to pseudocreatinines.

Copeptin was analyzed in 2024 using the frozen samples collected at inclusion. The samples were analyzed using a highly sensitive time-resolved amplified cryptate emission technology assay (B.R.A.H.M.S, KRYPTOR, AG, Hennigsdorf, Germany) at the Department of Diabetes and Cardiovascular Disease, Dept. of Clinical Sciences, Malmö, Lund University.

For 25-hydroxyvitamin D3, the samples were worked up according to the method previously described by Turpeinen et al. [22], after which derivatization with 4-phenyl-1,2,4-triazoline-3,5-dione was performed; they were finally analyzed by analyzed by high-performance liquid chromatography-electrospray tandem mass spectrometry in an inhouse assay. The used instrument was a TSQ Fortis Plus Mass spectrometer from Thermo Scientific (San Jose, CA, USA) combined with a Vanquish Binary Pump F and a Vanquish Split Sampler FT both from Thermo Scientific (Germering, Germany). The assay quality was assured by participation in the Vitamin D External Quality Assessment Scheme [23].

Data regarding fracture diagnoses was extracted from the Swedish national in- and out-patient register. All fracture diagnoses were requested from the time of the patients’ recruitment to the study until the start of 2022, and the ICD-10 (International Classification of Diseases) codes and time of diagnosis set by the patients’ physicians formed the extracted data.

### 2.3. Statistical Methods

All statistical calculations were performed using IBM SPSS version 29, and statistical significance was set at the *p* < 0.05 level.

Descriptive statistics were obtained by calculating the numbers and percentages of individuals with fractures across different groups. For continuous variables, the median and Q1 and Q3 were determined. Additionally, the number and percentage of each gender within various groups were calculated. The Variance Inflation Factor was calculated, as well as correlations between all included variables.

To assess baseline differences between groups, Mann–Whitney U tests were used for continuous variables and chi-square tests for categorical data. Dummy coding was performed for the variables’ gender and smoking: women were coded as 1 and men as 0, while smokers were coded as 1 and nonsmokers as 0. Cox regression was used to identify biomarkers for the risk of fractures, within both the control group and the patient group. To find potential confounders for the risk of fractures, we looked at previous research to establish which variables we know affect the risk of fractures in the general population. These potential confounders were included in the models to make sure that our biomarkers were independently associated with the risk of fractures. To evaluate model stability in the context of a limited number of events, the events-per-variable (EPV) ratio was calculated, and additional models using standardized biomarkers were constructed. The proportional hazard assumption was assessed by including time-dependent covariates (interaction terms with log(time)) in the Cox regression models. No violations of the assumption were observed. The relationship between the time of fracture and inclusion in the study was analyzed using Kaplan–Meier survival analysis, while differences were assessed using the Mantel–Cox test.

## 3. Results

### 3.1. Fractures and Baseline Information

During the time from recruitment in 2010–2013 to the follow-up in 2022, a total of 91 fractures were recorded, 59 of them among type 1 diabetes patients. Of these fractures, two were fractures of the cervical spine (S12) (zero among patients with type 1 diabetes), 12 were rib fractures (S22) (10 among patients with type 1 diabetes), four were fractures of the lumbar spine or pelvis (S32) (four among patients with type 1 diabetes), 60 were forearm fractures (S52) (34 among patients with type 1 diabetes), and 13 were femur fractures (S72) (11 among patients with type 1 diabetes).

As described in Table 1, 938 participants were included, of which 59.7% were women. The median age at inclusion was 54. In the patient group, there was a significant age difference between participants who sustained a fracture and those who did not (57 vs. 50, *p*-value < 0.001), but this was not observed in the control group (54.50 vs. 55.50). A significant difference in smoking status was observed between participants who sustained a fracture and those who did not within the control group (9 vs. 51, *p*-value 0.008). Furthermore, among participants with type 1 diabetes, significant differences in ApoB levels (median 0.92 vs. 0.87, *p*-value 0.014), ApoA1 levels (1.70 vs. 1.50, *p*-value 0.001), and GFR (88.00 vs. 90.00, *p*-value 0.029) were observed between those who sustained a fracture and those who did not. No other differences were found within the respective subgroups between participants who sustained a fracture and those who did not. There were several significant correlations between the included biomarkers and other variables; nevertheless, all the correlations were weak (rho <+/− 0.433). Only one VIF value was >2, namely, that for the patient/control variable (VIF = 2.103) in the entire population. When the patient and control groups were analyzed separately, no variable had a VIF above 2.

**Figure 2:**
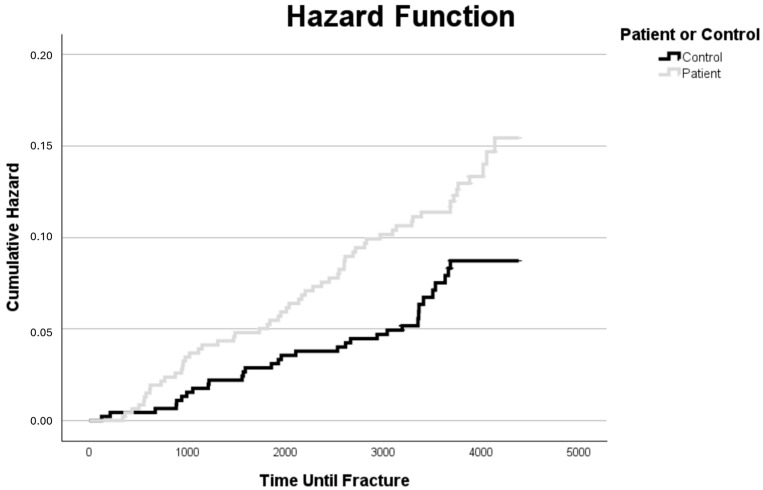
Cumulative hazard function for the risk of developing fractures over time. A cumulative hazard function showing the risk of receiving fractures over time for patients versus controls.

A Kaplan–Meier test was performed to compare the risk of fracture over time between the patient and control groups (Figure 2). The Mantel–Cox (log-rank) test demonstrated a statistically significant difference between the groups (*p* value 0.037), indicating a higher cumulative risk of fracture over time among patients with type 1 diabetes compared with controls. Cumulative incidence estimates were calculated at 2.5-year intervals. At 2.5 years, the incidence was 1.2% in controls and 2.0% in patients. At 5 years, it increased to 3.1% in controls and 4.0% in patients. By 7.5 years, the cumulative incidence reached 4.4% in controls and 8.2% in patients, and at 10 years, it was 8.8% in controls compared with 10.7% in patients. These findings demonstrate a consistently higher event probability in patients over time, highlighting the progressive divergence in risk between the two groups.

### 3.2. Biomarkers

Univariate Cox regressions were performed for the patient and control groups (Table 2). In the patient group, the risk of fracture was significantly associated with increasing age (HR 1.06, Cl 1.029–1.097, *p*-value < 0.001), ApoB (HR 4.947, Cl 1.627–15.038, *p*-value 0.005), and ApoA1 (HR 4.790, Cl 2.266–10.126, *p*-value < 0.001) and lower GFR (HR 0.986, Cl 0.973–0.999, *p*-value 0.034).

In the control group, the risk of fracture increased for those participants who were smokers (HR 2.678, Cl 1.239–2.678, *p* = 0.012).

Multivariable Cox regressions are demonstrated in Table 3, showing a step-by-step process including four models. The first model, model 1, included basic information; in model 2, all potential biomarkers were included. In model 3, HbA1c was included, meaning that only participants with type 1 diabetes were included. In model 1, the risk of fracture increased with age (HR 1.061, Cl 1.026–1.096, *p*-value < 0.001) within the patient group. Smoking significantly increased the risk of fracture within the control group (HR 2.581, Cl 1.173–5.680, *p*-value 0.018). Model 2 investigated whether ApoA1 and ApoB remained independently associated with the risk of fracture after adjustments for CRP, copeptin, GFR, smoking, age, and gender. Both ApoA1 (HR 4.186, Cl 1.839–9.527, *p*-value < 0.001) and ApoB (HR 8.201, Cl 2.249–29.902, *p*-value 0.001) remained significant predictors of fracture risk in the patient group. Increased age was also significantly associated with fracture risk in the group of type 1 diabetes patients (HR 1.033, Cl 1.015–1.094, *p*-value 0.006) but not in the control group. Smoking was only significant in the model restricted to the control group (HR 2.714, Cl 1.215–6.016, *p*-value 0.015).

After adjustments for all investigated variables (Model 3) (HbA1c, GFR, CRP, copeptin, age, smoking, cortisone treatment, physical activity, lipid-lowering medication, and gender), both ApoA1 (HR 4.290, CI 1.871–9.837, *p*-value < 0.001) and ApoB (HR 7.625, CI 1.995–29.138, *p*-value 0.003) remained independently associated with fracture risk in the patient group. Age was the only additional variable significantly associated with fracture risk in this model (HR 1.054, CI 1.016–1.094, *p*-value 0.006). Model 3 was restricted to participants with type 1 diabetes, as HbA1c results were available only for this subgroup.

Given the relatively wide confidence intervals observed for some predictors, potential model instability and the limited number of events relative to the number of covariates were assessed. In the patient group, 59 fracture events were observed, with thirteen predictors included in the primary multivariable Cox model (EPV ≈ 4.5). In the multivariable analysis including standardized ApoA1, ApoB, and all other variables from model 3, higher ApoA1 (HR 1.538, CI 1.220–1.939, *p*-value < 0.001) and ApoB (HR 1.601, CI 1.177–2.179, *p*-value 0.003) levels were significantly associated with an increased risk of fractures.

Finally, model 4 was fitted on the entire population, without stratification by group, with gender, age, smoking status, Vitamin D, ApoA1, ApoB, copeptin, and patient/control as the included variables. We found that the risk of fracture was independently associated with increasing age (HR 1.040, Cl 1.010–1.070, *p*-value 0.008) and higher ApoA1 (HR 3.241, Cl 1.591–6.604, *p*-value 0001) and ApoB (HR 2.901, Cl 1.049–8.022, *p*-value 0.040) levels. Furthermore, type 1 diabetes patients had a significantly higher risk of fracture when compared with control individuals (HR 2.257, Cl 1.320–4.209, *p*-value 0.004).

## 4. Discussion

In this study, we found that type 1 diabetes patients had an increased risk of fractures as compared with the general population. Furthermore, among patients with type 1 diabetes mellitus, fracture risk was associated with higher ApoA1 and ApoB levels, as well as increasing age. In the control group, fracture risk was associated with smoking.

In this study, all fractures occurring in the population were included, without any distinction between osteoporotic (fragility) fractures and other types of fractures. However, differences in the distribution of fracture types were observed between the groups. To further explore whether bone fragility in patients with type 1 diabetes reflects the same underlying mechanisms as fragility fractures in the general population, a comparison with an established definition of fragility fractures is relevant. Fragility fractures are typically defined as fractures of the hip, spine, pelvis, distal femur, proximal tibia, humerus, forearm, and multiple ribs according to the European Medicines Agency [24]. Notably, most fractures observed in our study fell within the typical spectrum of fragility fractures. This suggests that the increased fracture burden in patients with type 1 diabetes may, at least in part, reflect impaired bone strength similar to that seen in osteoporosis. However, in our data we do not know how the fractures occurred, and they were not classified by trauma mechanism (e.g., low vs. high energy). Meaning that we do not know that they actually were true fragility fractures. Additional research in this area would be of great interest, as it could have important clinical implications and provide further insight into the mechanisms underlying the increased fracture risk in individuals with type 1 diabetes.

### 4.1. Biomarkers for Predicting Fractures Among Patients with Type 1 Diabetes

We observed that higher ApoB levels remained independently associated with the risk of fracture, even after adjusting to other known risk factors of fractures. To the best of our knowledge, this association has not been previously demonstrated. Nevertheless, our results are in alignment with those of a previous study reporting a significant negative association between ApoB and BMD in the lumbar spine in a non-institutionalized US population [6].

We demonstrated herein that the risk of fracture was associated with a higher level of ApoA1, even after adjusting for other known risk factors of fracture. Previously, higher levels of ApoA1 were observed to be associated with an increased risk of osteoporosis in a general population [9]. This is in contrast to the observation that lower levels of ApoA1 are associated with the risk of osteoporosis in postmenopausal women with type 2 diabetes [8]. We found that higher ApoA1 and ApoB levels were associated with the risk of fractures in individuals with type 1 diabetes mellitus but not in the general population. The role of lipid metabolism and bone metabolism has been discussed previously and recently reviewed by Zhang et al. [25]. They argued that lipid metabolism and bone health are closely intertwined. According to previous research, high cholesterol, oxidized lipids, and fatty acid profiles contribute to a lower BMD. This is thought to be through different pathways wherein oxidized lipids activate pathways that promote adipogenesis over osteogenesis. Oxidized phospholipids and cholesterol also promote the release of RANKL from osteoblasts, which, in turn, enhances osteoclast differentiation and activity and bone resorption [25]. In addition to this, lipid disorders are known to trigger oxidative stress and inflammation through cytokines. The cytokines can act via RANKL on osteoblasts, stimulating the differentiation and activation of osteoclasts and thereby increasing bone resorption, ultimately leading to a lower BMD [25]. Higher plasma total cholesterol levels were reported to be positively associated with an increased risk of bone fractures, while lower high-density lipoprotein concentrations were linked to a higher risk of osteoporotic fractures [26]. In the mentioned study no significant association was found regarding the level of triglycerides and low-density lipoprotein [26]. Additionally, in another study no association was found between lower BMD and higher total cholesterol, triglycerides and low-density lipoprotein, or high-density lipoprotein and risk of fracture in subjects with type 1 diabetes [11].

Given that the mechanism underlying the increased fracture risk in patients with type 1 diabetes is unclear, it is difficult to draw firm conclusions about why ApoA1 and ApoB are associated with this risk. Many studies referenced to in this manuscript are limited to the general population; therefore the associations observed in the type 1 diabetes population must remain speculative. One hypothesis is that individuals with type 1 diabetes already exhibit altered metabolism, characterized by oxidative stress, episodes of hypo- or hyperglycemia, and low-grade chronic inflammation. When combined with the effects of lipids on bone, as described above, these metabolic disturbances may contribute to the associations observed between ApoA1, ApoB, and fracture risk in this population.

While some previous studies have linked ADH and bone metabolism [12,17], we did not find an association between the risk of fractures and copeptin concentrations. The previous studies connected hyponatremia and the risk of fractures, whereas our study specifically examined ADH levels in relation to fractures. Although hyponatremia and elevated ADH are connected, this does not necessarily imply that ADH concentrations alone are predictive of fracture risk, which may explain the discrepancies in findings. Unfortunately, we do not have data on natrium levels in our study population. It would, however, be interesting to investigate these connections in further studies. Furthermore, copeptin is primarily considered a marker of acute physiological stress and hemodynamic regulation, rather than a long-term biomarker. This may help explain the lack of association observed in our study between copeptin levels and fracture risk. While copeptin could reflect transient stress responses, our analysis did not include the copeptin levels at the time of fracture. Future studies with repeated measures or longitudinal assessments of stress-related biomarkers could further clarify their role in fracture risk among patients with type 1 diabetes.

We did not find a significant connection between HbA1c and the risk of fractures. This contrasts with previous studies suggesting that a higher level of HbA1c increases the risk of fractures [10,27], but it is important to highlight that Schneider et al. restricted their analysis to patients with type 2 diabetes. With findings similar to ours, however, Maddaloni et al. reported no significant association between HbA1c levels and fracture risk [11]. The fact that we did not find a connection with HbA1c could mean that the mechanisms behind the risk of fracture within this patient group are connected not to hyperglycemia but to something else entirely.

The number of fracture events was limited relative to the number of covariates (EPV ≈ 4.5), meaning that there is a risk of overfitting in the multivariable models. However, our findings were robust across several analyses. Univariate models for each biomarker showed hazard ratios consistent in direction and magnitude with those from the multivariable models, despite wide confidence intervals reflecting the limited number of events. Standardizing the biomarkers resulted in lower hazard ratios and narrower confidence intervals, improving interpretability while preserving statistical significance and the direction of effect. In conclusion, although the direction of the associations was consistent with univariable analyses and analyses using standardized variables, the magnitude of the effect estimates should be interpreted with caution, as they may be unstable and potentially overestimated.

We have shown that higher levels of ApoB and ApoA1 are significantly associated with fracture risk in individuals with type 1 diabetes. This suggests that the underlying mechanism differs from that of fracture risk observed in individuals without diabetes. However more research on the underlying pathways is necessary. Understanding this could be crucial in developing targeted strategies to foresee and prevent fractures in individuals with type 1 diabetes. Conventional fracture risk assessment tools may not be the best tools to evaluate risk in this group, highlighting the need for diabetes-specific biomarkers or adjusted algorithms.

### 4.2. Limitations

There are some limitations to this study. Perhaps most prominent is the fact that the blood samples were not obtained when the fracture occurred; instead, they were collected at the start of the study. Given that test results can fluctuate over time, they may not accurately represent the biomarker concentration at the time the fracture occurred.

In this study, we chose to exclude participants who had died prior to follow-up. This could, of course, have influenced the results. If a participant died before the end of the follow-up period, it is unknown whether they would have experienced a fracture; because of this, they were excluded from the study. This could have introduced a risk of bias, as the individuals who died likely had a higher burden of comorbidities and, consequently, a higher underlying fracture risk.

Furthermore, we have information about whether a fracture occurred or not but no information about the underlying mechanism. It might be more beneficial to either only focus on fractures related to osteoporosis or obtain more information about how the fracture occurred and only include the “low-impact fractures”. However, our aim with this study was to look at the overall fracture risk, not only at low-impact fractures. Further studies on the subject in regard to this would be beneficial.

This study’s limitations also included an unintentional gender imbalance with a prevalence of females and a failure to generalize our data to a non-Caucasian population, since our cohort was largely represented by Caucasian individuals. This lowers the external validity and generalizability of this study. Furthermore, additional confounders such as previous fractures would further strengthen our results.

A strength of this study is the long-term disease duration among participants with type 1 diabetes (>20 years); this enabled an assessment of long-term outcomes in type 1 diabetes. However, we also acknowledge that this may have introduced survivor bias, as individuals with more severe complications may not have survived. However, it should be noted that the study population reflects a clinically relevant group of patients living with long-standing disease, and the findings are therefore applicable to this population.

## 5. Conclusions

In this study, we showed that for patients with type 1 diabetes, the risk of fractures increases with higher ApoA1 and ApoB levels and age. We also confirmed that patients with type 1 diabetes have an increased risk of fractures. Because of the nature of this study, we cannot draw any conclusions about causality and can only theorize about the reasons behind our findings. However, our results suggest that the underlying mechanisms behind fractures in patients with type 1 diabetes differ from those observed in individuals without diabetes. ApoA1 and ApoB are rather cheap biomarkers to test for and are often used in a clinical setting. If the association could be confirmed in further studies, it could potentially be used in clinics to predict fracture events within this particularly exposed group. Replication in independent cohorts is necessary before clinical translation and to further analyze the underlying mechanisms. Further research linking lipid metabolism and skeletal health in type 1 diabetes would also be beneficial as it could determine whether the previously established methods of evaluating bone health insufficiently predict risk of fracture in this patient group.

## Figures and Tables

**Table 1:** Information regarding our population showing the number of participants in each group, as well as the various biomarkers and both the median and Q1 and Q3 for the continuous variables. Regarding physical activity, the responses were recoded as follows: 1 = never, 2 = less than once per week, 3 = 1–2 times per week, 4 = 3–5 times per week, and 5 = daily. HbA1c was only calculated for the patients with type 1 diabetes. The percentage was calculated using only valid cases within each column. Significant *p*-values are marked in bold.

	Total	Patients		*p*-Value	Controls		*p*-Value
		Fracture	No Fracture		Fracture	No Fracture	
Gender, female, N (%)	560 (59.70)	34 (57.63)	233 (56.28)	NS	21 (65.63)	272 (62.82)	NS
Age (years), median (Q1, Q3)	54 (46, 61)	57 (50, 61)	50 (43, 57)	**<0.001**	55(49, 63)	56 (45, 61)	NS
Smoking N (%)	123 (13.11)	5 (8.47)	58 (14.01)	NS	9 (28.13)	51 (11.78)	**0.008**
ApoA1 (g/L), median (Q1, Q3)	1.50 (1.32, 1.69)	1.70 (1.41, 1.87)	1.50 (1.33, 1.67)	**0.001**	1.18 (1.44, 2.01)	1.53 (1.29, 1.72)	NS
HbA1c (mmol/mol), median (Q1, Q3)	64.00 (57.00, 72.00)	64.00 (58.00, 72.00)	64 (57, 72)	NS	-	-	-
ApoB (g/L), median (Q1, Q3)	0.97 (0.83, 1.15)	0.92 (0.79, 1.11)	0.87 (0.77, 0.98)	**0.014**	1.34 (1.07, 1.38)	1.08 (0.96, 1.26)	NS
Copeptin (pmol/L), median (Q1, Q3)	4.76 (3.16, 7.60)	5.32 (3.34, 9.13)	5.20 (3.24, 8.13)	NS	4.96 (2.41, 9.15)	4.14 (2.92, 7.33)	NS
GFR (mL/min/1.73 m2), median (Q1, Q3)	89.00 (78.00 98.00)	88.00 (71.00, 95.00)	90.00 (77.00, 100.00)	**0.029**	88.00 (74.25, 96.50)	93.00 (81.75, 100.50)	NS
CRP (mg/L), median (Q1, Q3)	0.80(0.30, 2.30)	0.90 (0.30, 3.10)	0.95 (0.30, 2.50)	NS	1.15 (0.30, 4.85)	0.50 (0.30, 1.23)	NS
25(OH)D3 (nmol/L)	59.56 (46.25, 71.83)	58.92 (44.20, 70.00)	60.21(47.36, 71.66)	NS	61.88 (47.74, 73.54)	58.87 (45.31, 71.90)	NS
Cortisone treatment N (%)	29(3.10)	4 (6.78)	13 (3.14)	NS	2 (6.25)	10 (2.31)	NS
Lipid-lowering medication N (%)	291 (31.00)	35 (59.32)	217 (52.42)	NS	1 (3.12)	38 (8,78)	NS
Physical activity N (%)				NS			NS
1	36 (3.84)	4 (6.78)	12 (2.90)		1 (1.13)	19 (4.39)	
2	105 (11.19)	5 (8.47)	59 (14.25)		3 (9.38)	38 (8.78)	
3	234 (24.95)	12 (20.34)	102 (24.64)		4 (12.50)	116 (26.79)	
4	267 (31.56)	19 (32.20)	118(28.50)		13 (40.63)	146 (33.72)	
5	267 (28.546)	19 (32.20)	123 (29.71)		11 (34.38)	114 (26.33)	

Q1, Q3: 25th to 75th percentile; N: number; -: not applicable; NS: not significant.

**Table 2:** Univariate Cox regression regarding potential biomarkers for the risk of a fracture. Each Cox regression was constructed individually. Dummy coding was also performed for categorical variables, with smokers coded as 1 and nonsmokers as 0. Men were coded as 0 and women as 1. Treatment with cortisone or lipid-lowering medication was coded as 1, and participants who did not take such medication were coded as 0. Regarding physical activity, “never” was the reference variable. In the first row (1), “less than once a week” was coded as 1 and the rest as 0. In row 2, “1–2 times a week” was coded as 1. In row 3, “2–5 times a week” was coded as 1. Finally, in (4), “every day” was coded as 1. Significant *p*-values are marked in bold.

		N	HR	95% CI for HR	*p*-Value
Age					
	Controls	459	1.033	0.989–1.079	0.141
	Patients	473	1.062	1.029–1.097	**<0.001**
Gender					
	Controls	459	1.154	0.557–2.394	0.700
	Patients	473	1.050	0.627–1.760	0.853
Smoking					
	Controls	459	2.678	1.239–5.789	**0.012**
	Patients	473	0.568	0.227–1.421	0.227
ApoB					
	Controls	459	1.764	0.382–8.146	0.467
	Patients	473	4.947	1.627–15.038	**0.005**
HbA1c					
	Patients	473	1.006	0.983–1.029	0.630
ApoA1					
	Controls	459	2.233	0.631–7.908	0.213
	Patients	473	4.790	2.266–10.126	**<0.001**
Copeptin					
	Controls	459	0.992	0.942–1.044	0.750
	Patients	473	1.008	0.977–1.041	0.602
GFR					
	Controls	459	0.982	0.959–1.007	0.151
	Patients	473	0.986	0.973–0.999	**0.034**
CRP					
	Controls	459	0.981	0.852–1.129	0.788
	Patients	473	1.000	0.951–1.053	0.985
Physical activity					
1	Controls	459	1.391	0.145–13.378	0.775
2		459	0.623	0.0705–5.570	0.672
3		459	1.620	0.212–12.391	0.642
4		459	1681	0.217–13.021	0.619
1	Patients	473	0.269	0.072–1.003	0.051
2		473	0.363	0.117–1.124	0.079
3		473	0.493	0.168–1.448	0.198
4		473	0.475	0.162–0.162	0.176
Cortisone treatment					
	Controls	459	2.624	0.627–10.982	0.187
	Patients	473	2.004	0.726–5.531	0.180
Lipid treatment					
	Controls	459	0.327	0.045–2.398	0.272
	Patients	473	1.255	0.746–2.111	0.393
Vitamin D					
	Controls	459	1.005	0.987–1.023	0.612
	Patients	473	0.995	0.981–1.009	0.515

**Table 3:** Multivariable Cox regression regarding potential biomarkers for the risk of a fracture. Various multivariate Cox regression models were constructed for the biomarkers as stated. Dummy coding was performed, with women coded as 1 and men as 0. Dummy coding was also performed for the smoking variable, with smokers coded as 1 and nonsmokers as 0. Treatment with cortisone or lipid-lowering medication was coded as 1, and participants who did not take such medication were coded as 0. Regarding physical activity, “never” was the reference variable. Significant *p*-values are marked in bold.

		N	HR	95% Cl for HR	*p*-Value
Model 1 (gender, age, smoking, cortisone treatment, lipid-lowering medication, physical activity)					
Age					
	Controls	459	1.039	0.993–1.088	0.097
	Patients	473	1.061	1.026–1.096	**<0.001**
Smoking					
	Controls	459	2.581	1.173–5.680	**0.018**
	Patients	473	0.541	0.214–1.367	0.194
Model 2 (Model 1 + ApoA1, ApoB, CRP, GFR, copeptin, vitamin D)					
Age					
	Controls	459	1.033	0.981–1.089	0.220
	Patients	473	1.054	1.015–1.094	**0.006**
ApoB					
	Controls	459	0.841	0.139–5.082	0.851
	Patients	473	8.201	2.249–29.902	**0.001**
ApoA1					
	Controls	459	1.523	0.339–6.845	0.583
	Patients	473	4.186	1.839–9.527	**<0.001**
Smoking					
	Controls	459	2.714	1.215–6.061	**0.015**
	Patients	473	0.466	0.183–1.188	0.110
Model 3 (Model 1 + ApoA1, ApoB, CRP, GFR, copeptin, HbA1c)					
Age					
	Patients	473	1.054	1.016–1.094	**0.006**
ApoB					
	Patients	473	7.625	1.995–29.138	**0.003**
ApoA1					
	Patients	473	4.290	1.871–9.837	**<0.001**
Model 4: on the entire population (age, gender, smoking, cortisone treatment, lipid-lowering medication, physical activity, ApoA1, ApoB, copeptin, CRP, GFR, patient/control, vitamin D)					
Age		932	1.040	1.010–1.070	**0.008**
ApoB		932	3.025	1.057–8.658	**0.039**
ApoA1		932	3.241	1.591–6.604	**0.001**
Patient/control		932	2.357	1.320–4.209	**0.004**

## Data Availability

The original contributions presented in this study are included in the article. Further inquiries can be directed to the corresponding author.
